# Supplementary material for: Functional Role of Fatty Acid Synthase for Signal Transduction in Core-Binding Factor Acute Myeloid Leukemia with an Activating c-Kit Mutation
Source: Biomedicines. 2025 Mar 3;13(3):619. doi: 10.3390/biomedicines13030619 (PMC11939861; doi:10.3390/biomedicines13030619)
Supplement: Supplementary file 1 [file biomedicines-13-00619-s001.zip › biomedicines-3465284-supplementary.pdf]

Supplementary Materials and Methods

Materials

Kits

DC-Protein Assay Bio-Rad (Munich, Germany)

NucleoBond Xtra Midi Macherey-Nagel (Düren, Germany)

transfection reagents Lipofectamine, Gibco Thermo Fisher Scientific (Waltham, MA, USA)

P3000 Gibco Thermo Fisher Scientific (Waltham, MA, USA)

Super Signal West Dura

chemiluminescence substrate

Thermo Fisher Scientific (Waltham, Massachusetts)

Antibodies

panAKT #4685S rabbit monoclonal antibody Cell Signaling Technology (Beverly, MA, USA)

pAKT S473 #4060S rabbit monoclonal antibody Cell Signaling Technology (Beverly, MA, USA)

S6 #2217S rabbit monoclonal antibody Cell Signaling Technology (Beverly, MA, USA)

pS6 #2215S rabbit monoclonal antibody Cell Signaling Technology (Beverly, MA, USA)

MAPK #4695S rabbit monoclonal antibody Cell Signaling Technology (Beverly, MA, USA)

pMAPK #4377S rabbit monoclonal antibody Cell Signaling Technology (Beverly, MA, USA)

FASN #48357 rabbit monoclonal antibody Santa Cruz biotechnology (Heidelberg, Germany)

anti-mouse IgG HRP-linked antibody, #7076, Cell Signaling Technology (Beverly, MA, USA)

anti-rabbit IgG HRP-linked antibody, #7074, Cell
Signaling Technology (Beverly, MA, USA)

Bacterial strain

Xl1Blue Pierce Thermo Fisher Scientific (Waltham, MA, USA)

Vectors

PLKO.1-puro vector encoding FASN, PLKO.1 non-target (scrambled, SCR) shRNA were purchased from Sigma-Aldrich (Taufkirchen, Germany).

Inhibitors

TVB3166 was given kindly from Maxim Kebenko

Software

Microsoft Office 2007

Graphpad Prism: Version 8.2

AIDA Image Analyzer: Version 3.44

Zotero: 6.0.20

Methods

Culturing of cells

Cells were protected from contamination by working under a safety cabinet class II. Devices that came in contact with cells were autoclaved. Devices used under the safety cabinet were disinfected with a 70% solution of ethanol. Before usage, medium was warmed up in a 37°C water bath and trypsin was warmed to room temperature. PBS was kept at room temperature. Cells were cultivated in an incubator at 37°C with 5% CO2.

Thawing of cells

Cells were stored in 90% FCS and 10% DMSO in cryo tubes a nitrogen tank at -80°C. For

defrosting they were warmed in the water bath and quickly transferred to warm medium. DMSO was removed by centrifuging the cells for 5 minutes at room temperate at 1400rpm and removing the medium with the pipetting controller. Afterwards, the cell pallet was resuspended in 10ml of warm medium and transferred to a T75 cell culture bottle.

Passaging of cells

Cultures were maintained by the addition of fresh medium or replacement of medium or alternatively were established by centrifugation with subsequent resuspension at 3 x 105 viable cells/mL. Cell density was maintained between 3 x 105 and 3 x 106 viable cells/mL. Fresh medium was added every 2 to 3 days depending on cell density.

Freezing of cells

Cells were frozen when the density of cell was roughly 1.5 x 106 viable cells/mL. The cells were centrifuged at room temperature and 14000rpm for 5 minutes. The pallet was then resuspended in 90% FCS and 10% DMSO and immediately transferred to a cryo tube. The cryo tube was stored for 24hours in a freezing device before it was moved to the -80°C nitrogen tank.

Counting of cells

For evaluating cell concentrations, a Neubauer cell counting chamber was used. 10µl cell suspension was aliquoted and mixed with 10µl trypan blue solution and then pipetted between the cover slip and the chamber. Cells in the chamber were counted under the microscope. This was repeated three times per cell line. The mean was divided by dilution factor two. The number equals the number of cells times 10^4 per ml.

Incucyte Zoom, proliferation

For inhibitor treatment, Kasumi1 cells were plated into a 96-well plate with 10000 cells per well and incubated in 100µl RPMI 20% FCS 1% P/S per well. Cell confluence was measured by the IncuCyte Zoom imaging system (Essen Bioscience). After 24 hours 100µl of inhibitor

solution was added.

Kasumi1 SCR and FASN knock down cells were also plated into a 96-well plate (Greiner Bio one, Frickenhausen, Germany) with 10000 cells per well and incubated in 200µl RPMI 20% FCS 1% P/S and 1.5µg/ml puromycin.

Lentiviral knockdown of FASN

Transformation

100µl Xl1 blue bacteria were defrosted on ice and 100ng plasmid DNA was added. The solution was mixed carefully and incubated for 30 minutes on ice. Afterwards, the bacteria suspension was shock heated to 42°C for one minute and immediately put back on ice.

After adding 900µl warm LB medium, the solution was incubated at 37°C for 1 hour.

Thereafter, 150µl suspension was pipetted on LB Agar plates containing 100µg/ml Ampicillin for selection and spread out evenly with a spreader rod. After drying, the plates were incubated faced down over night at 37°C. The next day, colonies are picked with a sterile 10µl pipette tip and the tip was placed into the LB broth medium. Ampicillin was added to the medium with a final concentration of 100µg/ml. The bacteria were incubated at 37°C over night.

Plasmid prep

All reagents are part of the "NucleoBond Xtra plasmid purification Kit", unless otherwise stated. The bacteria suspension was filled into centrifuge tubes and centrifuged in the GSA rotor at 4°C and 5000rpm for 10 minutes. The pallet is resuspended in 8ml resuspension buffer (RES) and then incubated in 8ml lysin buffer (LYS) for 5 minutes after inverting 5 times. Afterwards, the neutralization buffer (NEU) was added and the solution was inverted until the blue color disappeared. The columns (NucleoBond Xtra Columns) were loaded with 12ml equilibration buffer (EQU) and linked to a filter holder each and then the suspension was filtered through. Columns and filters were washed in 5ml EQU and the filters were disposed. The filter holder was filled with 15ml washing buffer (WASH). The eluate was put into a 50ml tube each and 5ml elution buffer (ELU) was added. Then, each eluate was separated into 830µl aliquots and filled into 6 1.5ml tubes each with 580µl isopropanol. The

tubes were inverted and centrifuged at 13200rpm at room temperature for 5 minutes. Next, the supernatant was disposed and 200µl of 70% ethanol (this was not part of the NucleoBond Xtra plasmid purification Kit) were added, before the solution was centrifuged again at 13200rpm at room temperature for 5 minutes. As much supernatant as possible was removed and the DNA was dried out. Then, 20µl of sterile distilled H2O was added to each tube and the tubes were left at room temperature for 5 minutes. Afterwards, the tubes were whirled gently, and all 6 tubes were added together in one tube with 80µl of sterile H2O and whirled again. The DNA concentration was determined in the NanoDrop 2000c (Thermo Scientific) and the plasmid solution was frozen at -20°C.

Transfection of HEK293T

4 days before, HEK293T had been plated in one 10cm dish per knock down with 2x105 cells per dish. The day of the transfection, 2.5µg DNA of each vector was diluted in 20µl P3000 reagent and 8µg VSVG and gagPol. For each knock down, one tube containing 720µl OctiMEM and 30µl Lipofectamine were prepared and whirled. All the solutions were whirled shortly. Then, each OctiMEM and lipofectamine solution was added to one of the DNA solutions, inverted three times and incubated at room temperature for 10 minutes. In the meantime, medium of the HEK293T in the 10cm dishes was removed and replaced by 5ml DMEM 10% FCS without Penicillin/Streptomycin. Thereafter, the DNA solutions were added to the HEK cells and incubated for three hours. Meanwhile, 24 A and 24B cells were plated into 6 well plates with 3x105 cells per well in 2ml medium. At least one plate per knockdown and cell line was needed. After three hours of incubating, 2.5ml DMEM 10%FCS with Penicillin/Streptomycin is added to the HEK cells.

Transduction

24h after transfection, medium was removed from HEK293T using a 10ml syringe. After drawing up a 22µl filter was placed on the syringe before the virus supernatant was pushed into a 15ml tube. Next, 9ml DMEM 10%FCS/1%P/S was added to HEK cells. Then, the medium of the target cells was removed and 2ml virus containing HEK medium was placed onto the cells. 1ml DMEM 10%FCS/1%P/S was added. One well per cell line and plasmid was transducted. The other three wells in the 6 well plates were used as controls and only incubated with 3ml DMEM 10%FCS1%P/S. The rest of the virus supernatant was frozen at -

80°C. The procedure was repeated 48hours after transfection. 24hours later, the virus containing medium was removed from the target cells. The cells were then washed in 4ml PBS and 4ml DMEM 10% FCS 1% P/S containing 4μg/ml Puromycin was added for selection. 4ml DMEM 10% FCS 1% P/S containing 4μg/ml Puromycin was also added to the control wells. Selection was considered completed, when all cells in the control wells were dead.

Western Blot

Production of whole cell lysates using NP40 lysis buffer

The compounds Aprotinin, Sodium orthovanadate and Phenylmethylsulfonyl fluoride (PMSF) were added shortly before usage of the NP40 lysis buffer and from there kept on ice. The cells had been plated into 10cm dishes two days before with 5x106 cells per dish.

The medium was removed, and the cells were washed in 10ml cold PBS. Next, 1ml NP40 lysis buffer per dish was added and incubated on ice for three minutes. The cells were than scratched from the surface using a cell scraper and the solution was pipetted up and down ten times before being transferred to a 1.5ml reaction tube. The reaction tube was then centrifuged at 12000rpm and 4°C for ten minutes. After centrifugation the supernatant was transferred to another 1.5ml reaction tube and frozen at -80°C until used. The pallet was discarded.

Determination of the protein concentration using the DC protein assay kit

For determining the protein concentration of protein lysates, the DC protein assay kit is used. In alkaline environment, proteins react with copper and then reduce Folin reagent which leads to a blue color. The protein lysates were defrosted on ice and 5μl each were given into a micro plate as triplicates. For a standard curve, bovine serum albumin in concentrations of 0.125mg/ml, 0.25mg/ml, 0.5mg/ml, 0.75mg/ml, 1mg/ml, 1.25mg/ml and 1.5mg/ml were produced and given into a microplate with 5μl each as triplicates. Next, 25μl of a dilution of 2% reagent S in reagent A was added to each well. After adding 200μl of reagent B into each well, the micro plate was incubated at room temperature for 15 minutes. The micro plate was measured in a micro plate reader at adsorption modus, 750nm wavelength, 9nm range and 25 flashes. A linear standard curve was created and by using

the extinctions of the protein samples the protein concentration was calculated.

SDS-page

SDS-page was used to separate different proteins according to their size. After the gel tray was put together as described by the manufacturer, 13µl running gel was added, covered with 600µl isopropanol and incubated at room temperature for one hour. Afterwards, the isopropanol was displaced by water and emptied out carefully, before 5µl of stacking gel was added and 15 pocket cones were placed into the gel to generate pockets with 1.5cm length. The stacking gel was also incubated for at least one hour. The protein lysates were defrosted on ice and a diluted in NP40 lysis buffer to generate equal protein concentrations. The lysates were then diluted 1.3:4 in a dilution of 70% loading buffer and 30% dithiothreitol (DTT) and 70% loading buffer. The protein samples were cooked at 95°C for 4 minutes and shortly centrifuged to 13000rpm. Before applying 40-60µl of the samples to the gel, the cones were removed, air bubbles were removed with water and cathode buffer was added into the pockets of the stacking gel. In each gel, at least one pocket was filled with 4µl of protein marker. The gel tray was placed into the blot chamber, that had been filled with running buffer. The upper chamber was filled with cathode buffer. Finally, a voltage no higher than 120V was applied until the loading buffer bands reached the running gel and 175V until they reached the end of the gel.

Transfer and development

The gels were removed from the gel trays and gently washed in ddH2O. 6.5cmx14.5cm pieces of nitrocellulose membrane were humidified in ddH2O before usage. The transfer trays were placed into transfer buffer and the blot sandwich was built together. From outside to inside it consisted of two sponges, two 6.5cmx14.5cm sized Whatman paper pieces, the gel and the membrane in direct contact without air bubbles. Two sandwiches fit into one transfer tray. The transfer trays were then placed into the transfer chamber, which had been filled with transfer buffer until the blot sandwiches were covered completely. A voltage of 65V was applied to the transfer chamber for two hours. After opening the blot sandwiches, the gels were discarded, and membranes were washed in ddH2O for at least 2 minutes.

The colorful protein marker bands were marked with a pencil. The membranes were then stained in Penceau staining until protein bands were detected and cut down to useful sizes

or different pieces, according to the proteins that were focused. Penceau staining was removed by TBS-0.5% Tween and the membranes were placed in blocking solution and blocked at room temperature while gently shaking. After one hour, the blocking solution was discarded and washing solution containing the respective primary antibody in a dilution of 1:1000 was added. The membranes were than incubated over night at 8°C. The next day, the antibody solutions were removed, sodium azide was added in a concentration of 1:1000 and the solutions were stored at 4°C until further usage. The membranes were washed in TBS-0.5% Tween three times for 5 minutes. Subsequent, the suitable secondary antibodies were added as a dilution of 1:5000 in washing solution and incubated at room temperature. After one hour, the secondary antibodies were discarded, and the membranes were washed in TBS-0.5% Tween four times for 15 minutes. The membranes were developed using the LAS 4000 imager and Super Signal West Dura chemiluminescence substrate kit (Thermo Fisher Scientific)

Supplementary Figures

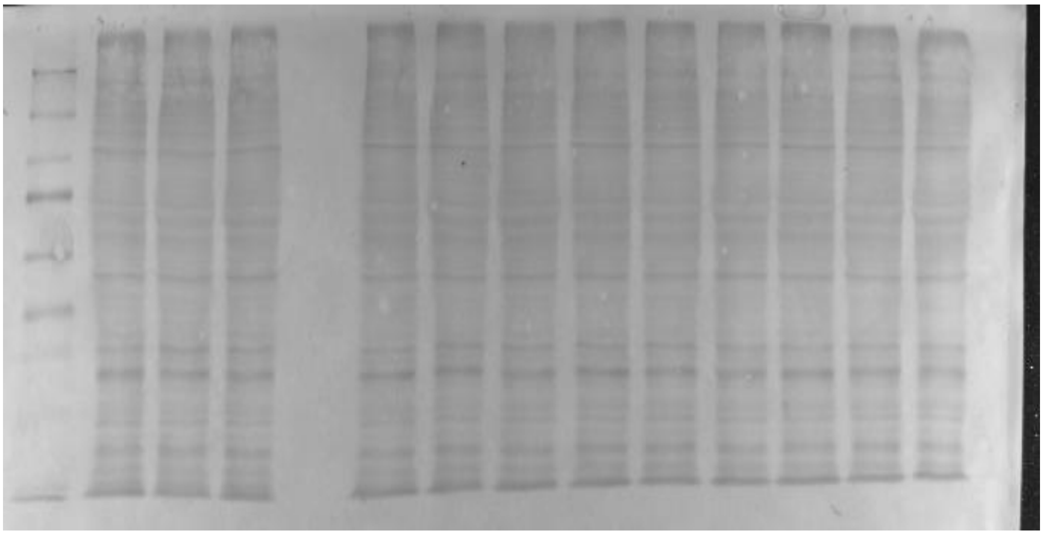

SUPPLEMENTARY FIGURE S1: shRNA-mediated knockdown of endogenous FASN in Kasumi1 cells (Ponceau staining).
FASN expression was detected in whole cell lysate by western blot. Kasumi1 FASN kd cell lines transduced with FASN shRNA (either with vector1 which is kd1 or with vector2 which is kd2) or Kasumi1 scr cell line transduced with non-targeting vector as a control for the baseline expression of FASN in Kasumi1 cell line. HSC70 was used as loading control for the quantification of FASN expression. The western blot is shown on the left and the statistic results on the right. Analysis is shown on the right side with y axis of fold change normalized to the expression level of FASN in scr. Each sample was loaded 3 times and analyzed by t test. Significance is presented in the graphs as * for $p<0.05$.

B

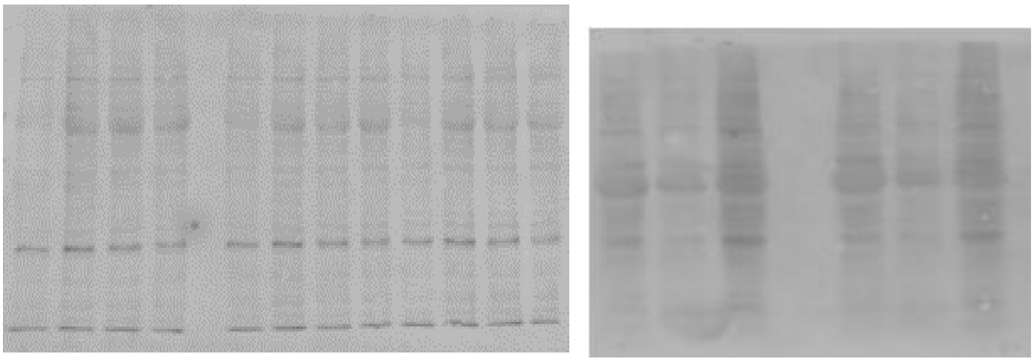

C

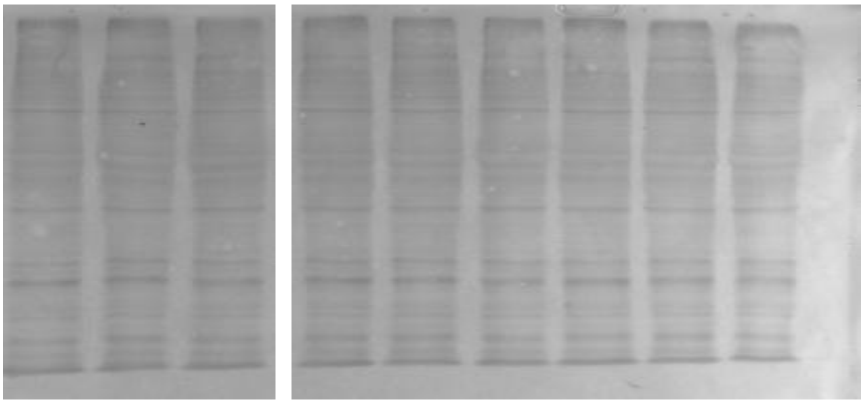

SUPPLEMENTARY FIGURE S2: Analysis of cKit signalling transduction in Kasumi 1 cells by western blotting (Ponceau staining).

A: Increased phosphorylation of S6 after knockdown of FASN in Kasumi1 cells, no significant regulation of Akt and MAPK detected in Kasumi1 cells with stable FASN-knockdown kd1 and kd2 and scr control cells. Samples were analyzed in technical triplicates. One of each of the three triplicates are shown on the left and the statistic results are shown on the right. Ponceau staining of the same membrane was used to demonstrate equal loading of protein lysates. Statistic bar charts used the optical density that were normalized to scr. Significance is presented in the graphs as * for p<0.05, ** for p<0.005, *** for p<0.001. B: Increased phosphorylation of S6 after treatment of Kasumi1 cells with TVB3166. Kasumi1 parental cells were incubated with 100nM TVB3166 for 0h, 4h, 8h, 24h and 48h. Protein lysates were analyzed for expression of phospho-S6 (pS6) and total S6 (S6) protein in technical duplicates and triplicates. Ponceau staining of the same membrane was used to demonstrate equal loading of protein lysates in each well which is shown beneath the western blots. Statistic bar charts used the optical density that were normalized to time 0h. Significance is presented in the graphs as **** = p<0.001. C: No significant regulation of Gli1 in Kasumi1 cells with stable FASN knockdown detected in technical triplicates. Ponceau staining of the same membrane was used to demonstrate equal loading of protein lysates. Statistic bar charts used the optical density that were normalized to scr control cells. Significance is presented in the graphs as *** = p<0.001

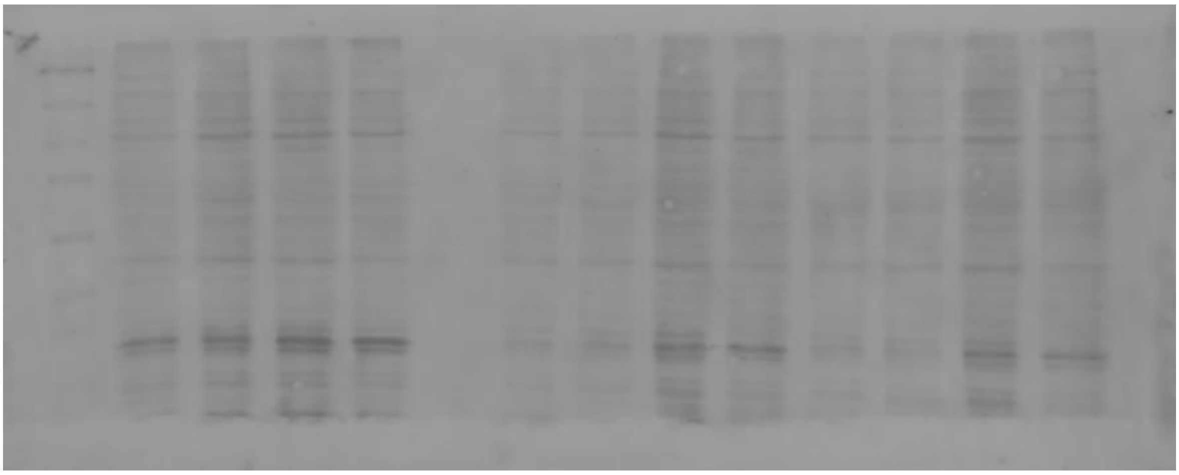

SUPPLEMENTARY FIGURE S3: Analysis of c-Kit N822K-mediated signal transduction in Baf3 cells by western blotting (Ponceau staining).
Ponceau staining of the membranes used for western blot analysis shown in figure 5 to demonstrate equal loading of protein lysates.

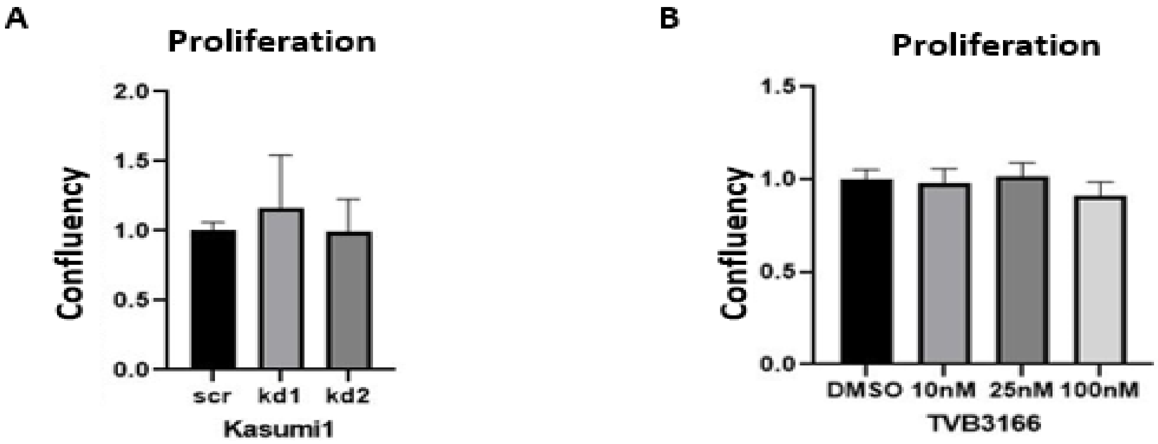

SUPPLEMENTARY FIGURE S4: Proliferation of Kasumi1 cells after knockdown of FASN or inhibition of FASN activity.

Proliferation of Kasumi1 SCR, Kasumi1 FASN knockdown1 (kd1) and Kasumi1 FASN knockdown2 (kd2) was analyzed by live cell imaging in an IncuCyte Zoom system (A). Proliferation of Kasumi1 parental cells was analyzing by IncuCyte live cell imaging system after treatment with either DMSO or different concentrations of FASN inhibitor TVB3166 (B). No significant changes could be detected.
